# Reducing inequities in maternal and child health in rural Guatemala through the CBIO+ Approach of Curamericas: 1. Introduction and project description

**DOI:** 10.1186/s12939-022-01752-y

**Published:** 2023-02-28

**Authors:** Mario Valdez, Ira Stollak, Erin Pfeiffer, Breanne Lesnar, Kaitlin Leach, Nina Modanlo, Carey C. Westgate, Henry B. Perry

**Affiliations:** 1 Curamericas/Guatemala, Calhuitz, San Sebastián Coatán, Huehuetenango, Guatemala; 2 Curamericas Global, Raleigh, North Carolina USA; 3 Independent Consultant, Winston-Salem, North Carolina USA; 4 Gillings School of Global Public Health, University of North Carolina, Chapel Hill, North Carolina USA; 5 Optum, SeaTac, Washington USA; 6 David Geffen School of Medicine at UCLA, Los Angeles, California USA; 7 Community Health Impact Coalition, New York, New York USA; 8 Health Systems Program, Department of International Health, Johns Hopkins Bloomberg School of Public Health, Baltimore, Maryland USA

**Keywords:** Maternal health, Child health, Community health, Primary health care, Community-based primary health care, Implementation research, Census-Based, Impact-Oriented Approach, Care Groups, Community Birthing Centers, Guatemala, Equity, Curamericas Global, Curamericas/Guatemala

## Abstract

**Background:**

The Curamericas/Guatemala Maternal and Child Health Project, 2011–2015, was implemented in the Western Highlands of the Department of Huehuetenango, Guatemala. The Project utilized three participatory approaches in tandem: the Census-Based, Impact-Oriented (CBIO) Approach, the Care Group Approach, and the Community Birthing Center Approach. Together, these are referred to as the Expanded CBIO Approach (or CBIO+).

**Objective:**

This is the first article of a supplement that assesses the effectiveness of the Project’s community-based service delivery platform that was integrated into the Guatemalan government’s rural health care system and its special program for mothers and children called PEC (*Programa de Extensión de Cobertura,* or Extension of Coverage Program).

**Methods:**

We review and summarize the CBIO+ Approach and its development. We also describe the Project Area, the structure and implementation of the Project, and its context.

**Results:**

The CBIO+ Approach is the product of four decades of field work. The Project reached a population of 98,000 people, covering the entire municipalities of San Sebastián Coatán, Santa Eulalia, and San Miguel Acatán. After mapping all households in each community and registering all household members, the Project established 184 Care Groups, which were composed of 5–12 Care Group Volunteers who were each responsible for 10–15 households. Paid Care Group Promoters provided training in behavior change communication every two weeks to the Care Groups. Care Group Volunteers in turn passed this communication to the mothers in their assigned households and also reported back to the Care Group Promoters information about any births or ﻿deaths that they learned of during the previous two weeks as a result of their regular contact with their neighbors. At the outset of the Project, there was one Birthing Center in the Project Area, serving a small group of communities nearby. Two additional Birthing Centers began functioning as the Project was operating. The Birthing Centers encouraged the participation of traditional midwives (called *comadronas*) in the Project Area.

**Conclusion:**

This article serves as an introduction to an assessment of the CBIO+ community-based, participatory approach as it was implemented by Curamericas/Guatemala in the Western Highlands of the Department of Huehuetenango, Guatemala. This article is the first of a series of articles in a supplement entitled Reducing Inequities in Maternal and Child Health in Rural Guatemala through the CBIO+ Approach of Curamericas.

## Background

The progress made globally over the past three decades in improving child and maternal health has been one of the great triumphs of public health. The number of deaths annually of children younger than 5 years of age (referred to hereafter as under-5 deaths) worldwide has declined by 58%, from 12.6 million in 1990 to 5.2 million in 2019 [1]. The number of maternal deaths annually worldwide has declined by 35%, from 451,000 to 295,000 during the same period [2].

Despite considerable progress, more work needs to be done. Of the 5.2 million under-5 deaths that occur each year, more than half (2.9 million) die within their first month of life, and most of these during the first 24 h of life [1]. An additional 2.0 million infants are stillborn each year [1]. Most of these deaths are from readily preventable or treatable conditions. Because we have the know-how and the resources to prevent these deaths, the ongoing mortality burden represents an urgent moral and public health challenge.

As the world moves ahead with Agenda 2030 to achieve the Sustainable Development Goals (SDGs), Universal Health Coverage, and Ending Preventable Child and Maternal Mortality [3], there is now a particular need to strengthen services and programs for those living in isolated, difficult-to-reach rural areas and for those who have been marginalized by unjust social and political circumstances. Ending Preventable Child and Maternal Mortality will involve achieving levels of child and maternal mortality that were achieved by the developed countries in the mid-twentieth century [4]. Given the notable disparities of child and maternal mortality that exist within low- and middle-income countries around the world, the global achievement of Agenda 2030 will require reduction in health disparities of all types, including ethnic and geographic disparities within countries, as called for by SDG 10 [5].

Health equity requires giving increased attention (i.e., funding, education, human resources for health, facilities) to those with the greatest health needs [6, 7]. Martin Luther King, Jr., in a speech in 1966 to the Medical Committee for Human Rights, proclaimed, “*Of all the forms of inequality, injustice in health care is the most shocking and inhumane*” [8]. Between countries and within countries, inequalities in health status can be greatly reduced through stronger health programs that encourage healthy behaviors and that make good quality, essential health services readily available. Addressing the social and physical determinants of health such as improved levels of education and increasing the availability of clean water and sanitation are also required. In spite of marked improvements in health programming and health status around the world, inequities are not diminishing as much as many countries and stakeholders had hoped [9] even though reducing inequities with the goal ultimately eliminating them has long been on the global health agenda [10].

### Guatemala, equity and health

Latin America is widely regarded as the region of the world with the greatest disparities in socioeconomic development [11, 12]. Compared to other regions of the world, Latin America has the most pronounced disparities in income [13] and in health status [14]. Its health care systems are deeply fragmented and segmented, with better-financed public systems available only for a small segment of the population composed of salaried people and their families [13, 15].

One of the important reasons for these disparities is the fact that there are 40 million Indigenous people throughout Latin America. They comprise a significant proportion of the populations of Mexico, Guatemala, Bolivia, Peru, Chile and Brazil [13]. Indigenous people have not benefitted to the same degree as non-Indigenous peoples in Latin America in terms of socioeconomic development [16]; they account for 8% of the population but 14% of the poor and 17% of the extremely poor in the region [16]. They continue to experience notable disparities in the population coverage of reproductive, maternal and child health interventions [17].

Guatemala is a lower-middle-income country in Central America with a population of 17.2 million people [18]. It is a multi-ethnic, multi-lingual, and multi-cultural country, where Indigenous (Maya, Xinka, and Garífuna) people account for 41% of the total population, the highest (along with Bolivia) of all the countries of Latin America [16]. However, according to Indigenous peoples’ representatives, the true figure is closer to 60% [19]. Most of Guatemala’s poor are Indigenous people of Maya descent who live in rural areas that are mostly mountainous and isolated; 75% of Guatemala’s Indigenous people live in poverty, more than twice the percentage of the non-Indigenous population [20, 21]. The country is also characterized as a male-dominated (*machisto*) society, leading to low levels of educational achievement and literacy for women, high levels of gender-based violence against women, and dependency on men [20].

Among the countries of the Western hemisphere, Guatemala has some of the greatest socioeconomic disparities [12, 21], health-related inequalities [14, 17, 22], and ethnic group inequalities in coverage of reproductive, maternal and child health interventions [17]. Guatemala has the highest wealth-related inequality of under-5 mortality in Latin America [22]. It also has the lowest level of government health spending in Latin America, with only 2% of the gross domestic product allocated to public health [23]. Guatemala’s Ministry of Public Health and Social Welfare (*Ministerio de Salud Publica y Asistencia Social*, or MSPAS) receives only 1% of the country’s gross domestic product to finance health services for 83% of the population [23].

Though Guatemala has made notable progress in reducing its under-5 mortality rate from 64 deaths per 1,000 live births in 1995 to 26 in 2018, it is still one of the highest in Latin America [1, 24, 25]. Guatemala’s national maternal mortality ratio (MMR) has declined gradually between 2000 and 2018 from 161 to 95 maternal deaths per 100,000 live births [2]. Inequalities in terms of health status and access to health care services are reflected in the following indicators: 61% of Indigenous children in Guatemala are stunted compared to 34% of the non-Indigenous population, and Guatemala’s Indigenous population has the highest prevalence of stunting among all the countries in Latin America with a significant Indigenous population [26]. Guatemala is also the country in Latin America with the greatest differential in stunting between the country’s Indigenous and the non-Indigenous population [26]. The difference in percentage of Indigenous children 0–59 months of age who were stunted compared to non-Indigenous children has persisted and remained substantial: 33% in 2008 and 27% in 2014 [26, 27]. Socioeconomic inequalities in childhood stunting have in fact increased between 1996 and 2016 [28]. A national study of maternal mortality in Guatemala found that Indigenous women represent 54% of the country’s reproductive age women but experience 71% of all maternal deaths [29].

This paper is the first of 10 in a supplement that describes an innovative approach to maternal and child health activities in three municipalities of Guatemala that are in the so-called “Triangle of Death” that suffered almost four decades of genocidal massacres, torture, sexual violence and crimes against humanity between 1960 and 1996, leading to more than 200,000 deaths at the hands of the military government’s counterinsurgency operations [30, 31]. This genocide was only one manifestation of centuries of racist discrimination, marginalization, and exploitation inflicted on the Mayan population by the Ladino (Mestizo/non-Indigenous) population, a methodical marginalization that exploited Maya labor, confiscated their land, and denied them the rights due to them under Guatemalan law, including their right to health care [32–34].

Poor health outcomes for the Indigenous rural people are a direct result of inadequate delivery of health services by the government and a manifestation of the historical marginalization of Indigenous Maya (including abusive and disrespectful treatment at government health facilities [35]). For example, while there are 25.6 skilled health workers per 10,000 population in urban areas, there are only 3 per 10,000 population in rural areas, where the vast majority of Maya live [23]. In our Project Area, the closest public referral hospital is more than a 4-h drive over dangerous mountain roads. This is also attributable to the government’s deliberate disinvestment in public services, especially health services, pursuant to the World Bank Sustainable Adjustment Goals that de-invest in developing country infrastructure [23].

**Fig. 1:**
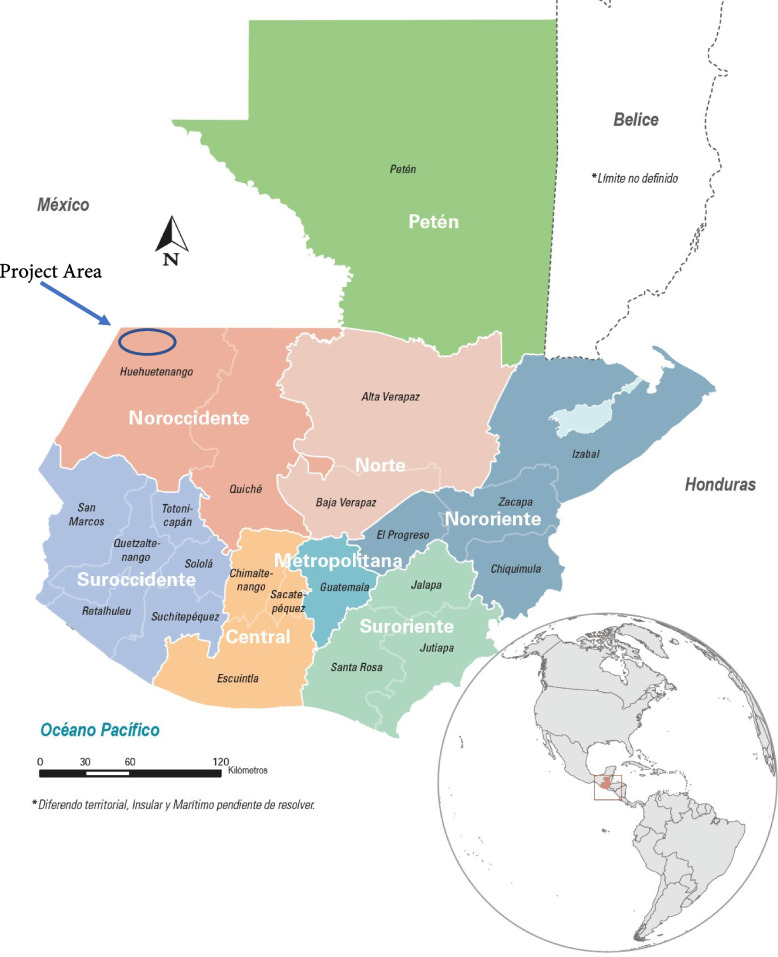
Location of the Curamericas/Guatemala Maternal and Child Health Project, 2011–2015. Source: Guatemala Demographic and Health Survey [36]

Curamericas/Guatemala implemented the Maternal and Child Health Project, 2011–2015 (hereafter referred to as the Project), in the Western Highlands of Guatemala in the Cuchumatanes mountains of the Department of Huehuetenango, Guatemala (Fig. 1). The goal of the Project was to improve the health and well-being of Indigenous Maya mothers and children living in an isolated mountainous area. Curamericas/Guatemala is a non-governmental organization established with the help of Curamericas Global. Curamericas Global has been supporting community-based primary health care programs since 1983 in Bolivia, Haiti, Kenya, Liberia, the United States, and Guatemala [37].

Due to the legacy of the genocidal violence inflicted on this population during the civil war, the population was extremely distrustful of outsiders and considerable effort was needed to win their trust, accomplished via meetings of Project staff with community leaders, presentations at community assemblies, involvement of community volunteers in the implementation of the Project, and regular contact between Project staff and the community.

The﻿ other papers in this supplement describe the implementation research methods [38] as well as the effects of the Project on population coverage of key maternal and child health interventions [39], nutrition [40], mortality [41], quality of maternity care at Community Birthing Centers [42], and women’s empowerment and well-being [43, 44]. The final two papers in the supplement concern assessments of staff and key stakeholders about the CBIO+ Approach [45] and a summary of the findings along with a discussion of the broader implications of this work [46].

## Methods

We first describe CBIO+ and its development. We then describe the Project Area, followed by a description of the Project and how it was implemented.

## Findings

### The CBIO+ Approach

The Project which is the subject of this series of papers relied on the CBIO+ Approach that combines the Census-Based, Impact-Oriented Approach (CBIO) with Care Groups and Community Birthing Centers (hereafter referred to as Birthing Centers). In the sections that follow, we define each of these elements and the process by which they came together as “CBIO+ ”.

#### CBIO

The Census-Based, Impact-Oriented (CBIO) Approach was developed in the 1980s on the Bolivian *altiplano* to address the challenge of achieving a sustainable improvement in the health of a rural Indigenous population of Aymara people in a resource-constrained setting [47]. The overarching goal of the CBIO Approach is health improvement at the population level with community partnerships playing a critical role. The approach is based on addressing two priorities: (1) the epidemiological priorities as determined from locally acquired surveillance data and (2) the community’s priorities as they themselves define them. CBIO brings health education and services to every doorstep on a regular basis. By registering births and deaths (vital events) at the time of regular visits to all homes, it is possible to monitor rates of mortality. Basic elements of the CBIO Approach are summarized in Table 1.

**Table 1:** Basic elements of the Census-Based, Impact-Oriented (CBIO) Approach

Overarching goals	1. Health improvement at the population level
Specific goals	1. Improvement of health in a specific, geographically defined population 2. Intermittent measurement of population health, with orientation of program priorities toward health improvement 3. Establishment of partnerships between communities and health-oriented program(s), essential for achieving maximal success in health improvement
Guiding principles	1. Diagnosis of epidemiological priorities, essential in order for the health practitioner to “prescribe” an effective “treatment” (the diagnosis and the prescribed treatment may change over time as health conditions change over time and as effective treatments change over time) 2. Use of locally acquired surveillance data (best obtained through visitation of all households or a sample of households), the most desirable approach to defining epidemiological priorities and to measuring changes in the level of health in the population over time 3. Choosing the right interventions and strategies for implementing these interventions (especially those that involve behavior change), aided by formative research techniques (these techniques are also useful in identifying community-perceived health priorities) 4. Identifying and responding to community health priorities, essential for building a partnership and trust 5. Routine contact with every household, required to build trust, achieve high coverage of services, and obtain optimal surveillance data (including vital events)
Initial steps (in a pilot area)	1. Development of a relationship of trust between the health practitioner and the community 2. Definition of the community (geographic boundaries, number and location of inhabitants) 3. Exploratory and then pilot planning and program implementation 4. Definition of community priorities
Definitive steps (in the complete program area)	1. Determination of the most frequent, serious, readily preventable or treatable causes of sickness, disability, and death, their underlying causes (through formative research), and those persons at greatest risk 2. Determination of the health priorities as defined by the community members themselves 3. Establishment of program priorities based on epidemiologically defined and community-defined priorities 4. Development of a work plan based on the program priorities and the resources available 5. Implementation of the program 6. Monitoring of progress on a regular basis and evaluation of the program periodically, followed by a community re-diagnosis (after 3–5 years)

This information has been adapted from Perry and Davis [48]

As implemented by Andean Rural Health Care (now called Curamericas Global) in Bolivia, first on the Northern Altiplano, then in the Cochabamba Valley, and later in a peri-urban population of the town of Montero in the tropical lowlands near the city of Santa Cruz, the implementation of the CBIO Approach achieved high coverage of key interventions and a reduction in the under-5 mortality by one-half [49, 50]. Three decades of CBIO implementation by Curamericas Global’s partner in Bolivia, *Consejo de Salud Rural Andino/Montero *(or CSRA/Montero) has led to an under-5 mortality rate at present that is less than half that of the United States – 3 versus 7 deaths per 1,000 live births [51].

#### Care Groups

The Care Group Approach is a pedagogic model for achieving behavior change at the household level that utilizes cascaded learner-empowered participation to actively engage participants in the learning process. Its lessons are designed for non-literate audiences and teachers. The Care Group model was developed in Mozambique by the international non-governmental organization (NGO) World Relief [52]. A Care Group consists of 5-12 female volunteers who are each responsible for 10–15 households, depending on the local geography. The Care Group meets every 2–4 weeks with a paid Promoter who teaches them a health promotion message to share with their neighbors. At the subsequent meeting, the facilitator teaches them a new message and the Care Group Volunteers report pregnancies, births and deaths to the facilitator [53]. A broad body of experience and evidence from implementation by many organizations throughout the world now supports the effectiveness of the Care Group Approach in achieving household-level behavior change resulting in high coverage of key interventions for maternal and child health as well as in improvements in child nutrition and under-5 mortality [54–57].

Curamericas piloted the combined CBIO and Care Group methodologies from 2002 to 2007 in Guatemala and later in Liberia between 2008 and 2013. These pilots demonstrated that Care Group Volunteers could report vital events and achieve high population coverage of evidence-based interventions for improving maternal and children health [58, 59].

#### Community Birthing Centers

Curamericas/Guatemala introduced Community Birthing Centers in response to the strong cultural practice of home births attended by traditional birth attendants (*comadronas*) and the lack of a physically and culturally acceptable and affordable alternative. At the time of the development of the first Birthing Center*,* the MSPAS operated only three clinics offering maternal/newborn care in the Project Area, and these were very distant for most of the population and functioned only during the daytime Monday through Friday. Many women found the care provided there to not be culturally appropriate. The only alternative was a hospital in the city of Huehuetenango four hours or more away, and this was unaffordable for most.

Curamericas/Guatemala established the first Birthing Center in the small town of Calhuitz in the municipality of San Sebastian Coatán in 2009. The facility provided a safe and clean space where a mother could come and deliver with a skilled birth attendant (a nurse or auxiliary nurse), assisted by a *comadrona* of the mother’s choice*.* It also provided a home-like atmosphere with a traditional Maya kitchen. Traditional birthing positions and rituals were encouraged. A traditional sweat lodge (*chuj*) where the mother could go after the delivery was also available.

A system of emergency transport was developed that could be activated if a complication arose that needed transfer to the hospital in the city of Huehuetenango. A supervising obstetric nurse was available for consultation in person or by phone as was the Curamericas/Guatemala Country Director (MV), who is an obstetrician. The community was involved in the construction and supervision of the Birthing Centers*.* This approach gradually proved to be feasible and popular, and led to a growing utilization of services as well as the construction of three additional Birthing Centers by 2015. A description of the functioning of these facilities and documentation of the early uptake in utilization of these Birthing Centers and their acceptance by *comadronas* has previously been reported [60].

### The Project: the Curamericas/Guatemala Maternal and Child Health Project, 2011–2015

Curamericas Global has been working with Curamericas/Guatemala since 2002 to address the challenges of improving maternal and child health in the Cuchumatanes mountains. This collaboration began when Dr. Mario Valdez, who had been working in this area since 1994 first as an MSPAS physician and later with Catholic NGOs in the area (including implementation of the *Seguro Medico Campesino* program), began to formulate ideas about how a census-based program of services delivered to households might be developed in this challenging area. This early work provided the vision for the government’s *Programa de Extensión de Cobertura* (PEC), or Extension of Coverage Program, described below. Dr. Valdez learned of the CBIO Approach developed by Andean Rural Health Care (as Curamercas Global was called at the time) and invited a representative to visit Guatemala. This led to preliminary activities and then a successful child survival project funded by the United States Agency for International Development (USAID) and its Child Survival and Health Grants Program in 2002. This project featured the combination of the CBIO methodology with the Care Group Approach. It was, in fact, one of the first Care Group projects outside of those initiated by World Relief and the first in Latin America. Thomas Davis, then working with Andean Rural Health Care, had visited the World Reliefs’s original Care Group project in Mozambique and became enthusiastic about the potential of the approach in Guatemala. This USAID grant came to a successful conclusion in 2007. Four years later (2011), Curamericas Global, working with Curamericas/Guatemala, was again successful in obtaining another highly competitive child survival grant for the Project whose evaluation is reported in this series of papers.

### Project Area

**Fig. 2:**
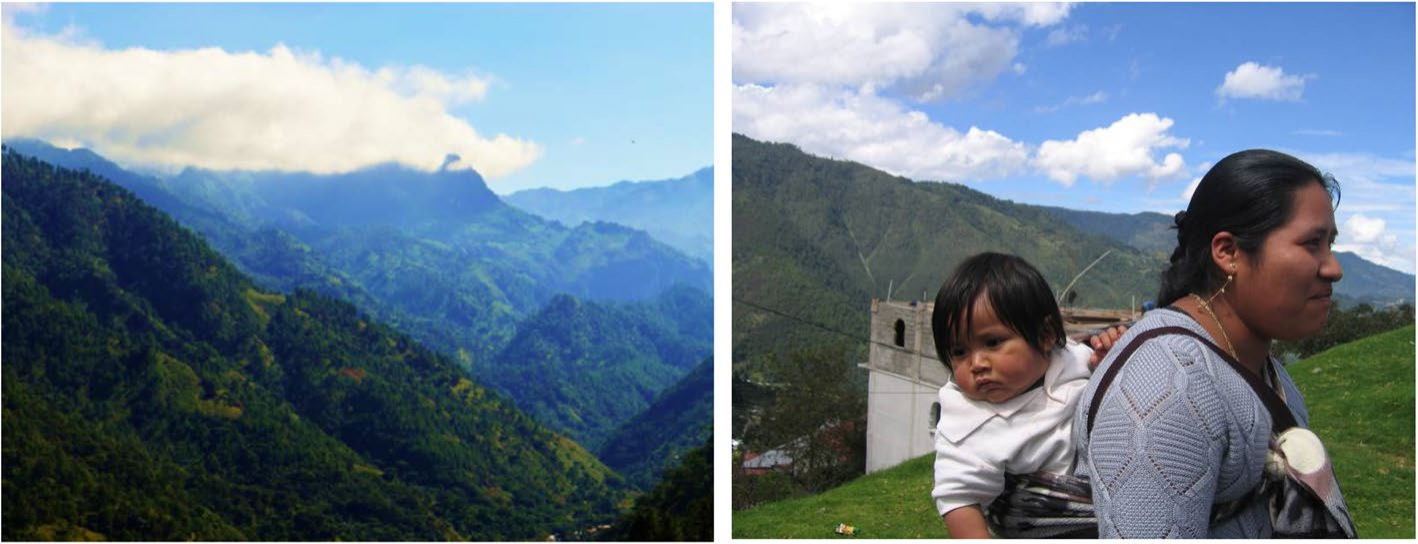
Typical view of the Project Area (left); mother and child living in the Project Area (right)_. Source: Mario Valdez

The Project was implemented in three municipalities (districts) of the Department (state) of Huehuetenango: San Sebastián Coatán, Santa Eulalia, and San Miguel Acatán (Fig. 1) from 1 October 2011 to 30 September 2015. These municipalities are in the Cuchumatanes mountains on steep terrain at an altitude of 7,000 to 9,000 feet (Fig. 2). Many communities have been accessible by road for only the past two decades, and many other communities are still accessible only on foot.

The Project Area, with a population of approximately 98,000, consists of 180 communities that are overwhelmingly populated by one of several Maya ethnic groups. In the municipality of San Sebastián Coatán, the Chuj ethnic group is predominant. In the municipality of San Miguel Acatán, the Akateko ethnic group is predominant. In the municipality of Santa Eulalia, the Q’anjob’al ethnic group is predominant. The dialects of Akateko and Q’anjob’al are mutually intelligible, but Chuj is unintelligible to Akateko or Q’anjob’al speakers.

The nearest public hospital was in the city of Huehuetenango, 4–5 h away. Private hospitals were located at the foot of the Cuchumatanes mountains in Nentón and Jacaltenango, about 1–2 h from the Project site, but no surgical services were available in Nentón and few of the Project inhabitants could afford to obtain care at these facilities. The MSPAS had only one physician posted in the Project Area. The health center in San Miguel Acatán had one graduate nurse and two auxiliary nurses working in it, but it had only a small laboratory and no x-ray facilities. In addition to this, there were six health posts in the Project Area, each staffed by an MSPAS auxiliary nurse with no services on the nights or weekends. Some of the staff were from outside the area and did not speak any of the local languages. The postings by MSPAS in these remote areas are widely considered to be “punishment posts” for MSPAS staff who had “gone astray” for some reason, and those who received appointments there often made major efforts to obtain appointments in more convenient locations as soon as possible. Many of them were often away from their posts (personal communication with numerous MSPAS staff). However, most of the lower level staff (e.g., auxiliary nurses) were from the local area and were fluent in the local language.

There were no physicians or nurses working as independent practitioners in the Project Area outside of the Curamericas Project staff. There were various traditional practitioners in the Project Area, consisting of traditional midwives (*comadronas*), and traditional healers (*curanderos*). The government’s PEC program staff consisted of two Nurse Supervisors, 10 Ambulatory (mobile) Nurses, seven Educators, seven Institutional Facilitators, and 25 Community Facilitators. Mobile health teams visited each community monthly to provide primary health care services. In the Project Area, PEC was financed by the MSPAS through a contract with Curamericas/Guatemala and another non-governmental organization, ADIVES (*Asociación de Desarollo Integral de Vida y Esperanza*). The PEC program is described further below along with further details about Project activities.

Based on information obtained from MSPAS at the outset of the Project, there were 47,657 direct beneficiaries of Indigenous Chuj, Akateko, and Q’anjob’al Maya people (32,330 women of reproductive age who were 15–49 years of age, and 15,327 under-5 children). The beneficiaries included infants 0–11 months of age (6.5% of beneficiaries), children 12–23 months of age (8.5%), children 24–59 months of age (16.0%), and women of reproductive age (69.0%) (Table 2).

**Table 2:** Beneficiary population in the Project Area by municipality and demographic group

Beneficiary population	Municipality/population			Total (98,341)
	**San Sebastián Coatán** **total population: 21,945**	**San Miguel Acatán** **total population: 30,977**	**Santa Eulalia** **total population: 45,419**	
Infants: 0- < 12 months of age	632	1,043	1,314	2,989
Children: 12- < 24 months of age	645	881	1,232	2,758
Children: 24- < 60 months of age	2,684	2,479	4,417	9,580
Children: 0- < 60 months of age	3,961	4,403	6,963	15,327
Women: 15- < 50 years of age	7,445	9,113	15,772	32,330
Total number of mothers and children	11,406	13,516	22,735	47,657

These three municipalities had some of the worst human development indicators in the country. In 2009, the three municipalities averaged 44 under-5 deaths per 1,000 live births and 681 maternal deaths per 100,000 live births according to MSPAS reports. According to data obtained from a baseline household survey in the Project Area in 2012 [61], the median level of education among mothers of children 0- < 24 months of age was only 3 years, 98% preferred to speak their native Maya language (Chuj, Akateko, or Q’anjob’al), and fewer than half were able to communicate in Spanish.

Household conditions were often unsanitary, drinking water was often contaminated, and handwashing was infrequent, all contributing to frequent episodes of childhood diarrhea. Childhood pneumonia was also frequent, exacerbated by the altitude, the chilly climate, and exposure to smoke from cooking indoors. At the outset of the Project, the great majority of births in the Project Area took place in the home and were attended by *comadronas*. Based on the January 2012 Project baseline knowledge, practice and coverage (KPC) survey findings, 89% of recent births had been attended by a *comadrona* at the mother’s home. Impeding health facility deliveries as well as proper care-seeking for sick children were the following: (1) long-distances (in terms of travel time) to MSPAS health facilities over the area’s rugged mountain terrain, requiring significant investments in both time and scarce financial resources; (2) traditions encouraging home deliveries and use of herbs and traditional healers and (3) disrespect and abuse experienced by Maya women at MSPAS health facilities, including refusal or an inability to provide culturally acceptable services in their language (Alma Dominguez, personal communication, 2012), a phenomenon documented by others in the Western Highlands of Guatemala, including Huehuetenango [35].

### Project partners

Curamericas Global is an NGO that was founded in 1983 as Andean Rural Health Care. It was created with the goal of establishing primary health care programs that were responsive to the health needs of the community, that improved child survival, and that built national and local capacity of health service providers. Initially, operations were based on the Northern Altiplano of Bolivia, which is home to Indigenous Aymara villagers. Curamericas Global initiated its programs in Guatemala in 2002 [37]. Today, CBIO remains the core approach for its programs in Bolivia, Guatemala, Haiti, and Kenya.

Curamericas Global has worked steadily towards a vision of sustainability which includes developing strategic partnerships with local and other stakeholders in order to establish integrated effective local rural health systems. Curamericas/Guatemala, founded by Dr. Mario Valdez as a Guatemalan NGO, is the implementing partner in Guatemala.

Curamericas/Guatemala carried out implementation activities through local staff, most of whom were Maya and spoke the local languages. Curamericas Global provided funding, training, technical assistance, and monitoring and evaluation (M&E). Curamericas Global backstops (Erin Pfeiffer and Ira Stollak) as well as trainers and consultants contracted by Curamericas Global provided capacity-building, technical assistance, and monitoring and evaluation (M&E) guidance.

MSPAS coordinated its services and exchanged information, primarily through staff at local clinics and health posts as well as through the government offices in the three municipalities where the Project operated. MSPAS also contracted with Curamericas/Guatemala to implement the PEC Program (described below) in the municipalities of San Sebastián Coatán and San Miguel Acatán. Two senior-level MSPAS staff members served as members of the Implementation Research Technical Advisory Committee. The three municipal governments contributed land for the construction of the Birthing Centers*.* At the grassroots level, the beneficiary communities, their leaders and especially mothers were essential partners, providing volunteer “sweat equity,” and feedback. The USAID Guatemala Mission provided ongoing advice and feedback to ensure that the Project was aligned with the strategic objectives of the Mission. Funding from the Ronald McDonald House Charities supported the construction, operation, and staffing of the Birthing Centers*.* The US-based NGO, Medicines for Humanity, provided a secure and reliable source of oxytocin for the Birthing Centers as well as medicines for a small pharmacy at each Birthing Center*.* Further details on these partnerships are provided below.

### Project description

The Project aimed to improve maternal and child health and nutrition and reduce maternal and under-5 mortality through community mobilization, capacity building, development of emergency transport systems, and achievement of high levels of population coverage of evidence-based interventions. The overriding goal was to integrate the Project with existing MSPAS services to create a coherent local rural health care system that addressed community and epidemiological priorities. The three components of the Curamericas/Guatemala CBIO+ Approach and an MSPAS program (PEC) were integrated to increase access, demand, and quality as well as to improve equity and enhance sustainability.

We included indicators of women's empowerment in the evaluation research because considerable anecdotal experience with Care Group implementation elsewhere had suggested that this approach empowered its participants and fostered social capital. Other Care Group projects had noted increased participation of women in family health-related decision-making and in community affairs.

**Fig. 3:**
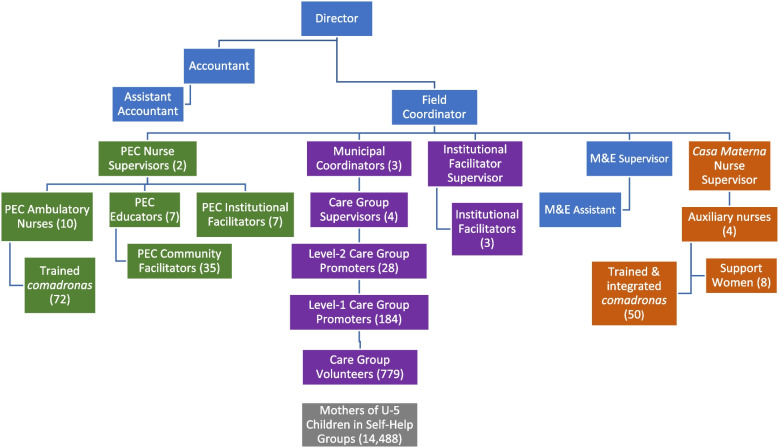
Curamericas/Guatemala Child Survival Project Organogram at the outset of the Project. Note:﻿ PEC: *Programa de Extensión de Cobertura* (Extension of Coverage Program). The Ministry of Public Heath and Social Welfare closed the PEC program at the onset of Year 4 of the Project and all of the PEC staff had to be terminated

Figure 3 provides the Project’s organizational and staffing structure, including the component funded through the PEC Program. The Project staffing arrangement consisted of three teams, one for each of the three municipalities in the Project Area. The work in each municipality was supervised by a Municipal Coordinator. Supporting them was a Care Group Supervisor in each municipality to supervise the Care Group activities. She supervised 8–10 Level-2 Care Group Promoters (called *Educadoras en Salud*). There were 28 of these total. These Level-2 Care Group Promoters supervised six or so Level-1 Care Group Promoters (*Facilitadoras Comunitarias*) who each oversaw and supported one or two Care Groups. There were a total of 184 Level-1 Care Group Promoters and 184 Care Groups.

Of the 184 Level-1 Promoters, 149 were funded by the Project and 35 by PEC. These were literate community collaborators, usually female, who worked part-time and received a modest monthly stipend (approximately US$35) for their work of training and supporting the Care Groups and collecting and conveying vital events data from their Care Group Volunteers. All were selected by their communities. In addition to their work in training Care Group Volunteers, they assisted with health emergencies and often accompanied patients to the hospital when that was necessary, providing translation from Spanish to the Maya dialect and other support as needed. Care Group members (called Care Group Volunteers, or *Comunicadoras en Salud*) were each responsible for 10–15 neighbor women.

The Level-2 Promoters were the backbone of the field staff. They each had an assigned territory of 5 to 8 communities, and in those communities they: (1) initiated the CBIO mobilization, census taking and mapping, and the Community Register; (2) trained and supported a Level-1 Promoter in each community to in turn train and support the community’s Care Group Volunteers ﻿and to receive and manage vital events data; (3) conducted routine home visitations for growth monitoring, vitamin A supplemention and deworming, promoting antenatal care and post-partum checks, family planning promotion, and follow-up for sick or undernourished children; (4) collected, organized, and analyzed monitoring and vital events data for their communities and relayed the data to the M&E staff for aggregation with Project data; and (5) coordinated with community leadership to disseminate and discuss the community’s health data at *asambleas* (community meetings) and to jointly plan community responses. These municipal field teams were supported by a two-person M&E team and a two-person accounting/fiscal management team, both partly funded by PEC.

In addition, in each municipality an Institutional Facilitator (a nurse) maintained the vital events registers and performed verbal autopsies. A Project Institutional Facilitator Supervisor oversaw the work of these three Instititional Facilitators.

As shown also in Fig. 3, parallel to the Curamericas/Guatemala staff structure were (1) the PEC staff, with two Nurse Supervisors overseeing ten Ambulatory Nurses, seven Institutional Facilitators, and seven *Educadoras*; and (2) the staff of the three Birthing Centers (an auxiliary nurse and two support women for each of the Birthing Centers*,* all of whom were supervised by an obstetric nurse supervisor). Overseeing all this was a Field Coordinator and the Curamericas/Guatemala Director Dr. Mario Valdez.

**Fig. 4:**
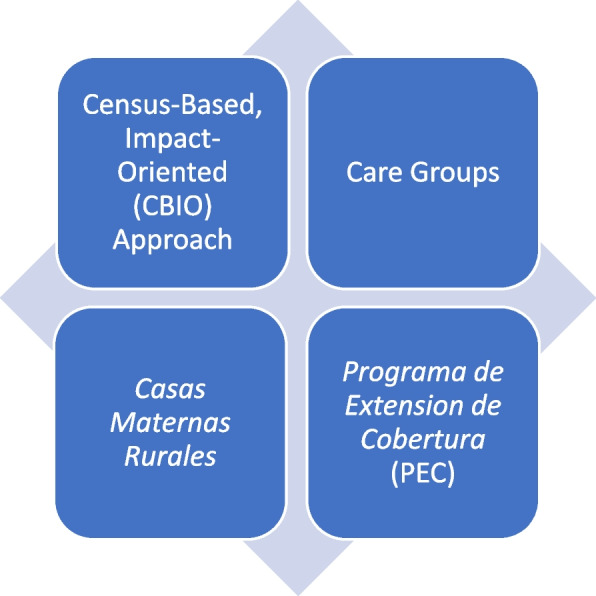
The four cornerstones of the primary health care system in the Project area

The Project complemented PEC by focusing on community-based preventive education, community mobilization, and linking communities to the PEC program to create an effective and comprehensive rural primary health care system with four cornerstones: CBIO, Care Groups, the Birthing Centers, and PEC (Fig. 4). Demand for health services was created via the Care Group methodology through behavioral change communication, health education, and community consciousness-raising about epidemiological priorities. This demand was fulfilled, when possible, by the Birthing Centers and the PEC program. Household monitoring through the CBIO methodology made it possible to identify those in need of health services and to assist women and children in obtaining the services they needed. The vital events surveillance of CBIO made it possible to monitor actual impact on maternal and child mortality.

Each component of the Project’s CBIO+ Approach – CBIO, Care Groups, and Birthing Centers* –* were described earlier. Here we provide additional details about their implementation in the Project area.

#### Application of the CBIO Approach in the Project Area

CBIO was implemented in the Project Area following the guidelines of two implementation manuals: a generic one in English [62] and one in Spanish developed specifically for this Project [63]. The CBIO process began slowly with the “generation of trust” stage during which the Project staff and community became acquainted, the community began to understand the purposes and methods of the Project, and the Project staff began to earn the community’s trust by fulfilling promises and exhibiting honesty and respect.

A key tool for this process is the open community assemblies (*asambleas*). In Guatemala, these are traditional community meetings open to all during which, in this first stage, mutual acquaintance and trust could begin to be built. The steps were as follows:

1. Mobilizing communities through local leaders to cement good relations and establish trust as well as to secure community buy-in and ownership
2. Conducting censuses and participatory community health assessments in each community leading to a Community Diagnosis (*Diagnóstico Comunitario)* that focused on the community’s health priorities
3. Drawing community maps, enumerating households, and creating a Community Register of every beneficiary by household
4. Establishing Community Health Committees and developing Community Health Plans with community members based on both epidemiologically-derived and community-perceived health priorities
5. Using the Community Registers to monitor coverage of health services to Project beneficiaries and record vital events
6. Making routine visits to all homes, with more frequent visits to those homes with special needs
7. Utilizing a continuous health surveillance system that allows staff to tailor service delivery and engage in continuous quality improvement

The health surveillance included ongoing registration of all births and deaths occurring in the communities, with verbal autopsies completed for all under-5 and maternal deaths to ascertain causes, and the calculation of under-5, neonatal (0–<28-day), post-neonatal (1–<12-month), and child (12–<60-month) mortality rates and maternal mortality ratios to monitor impact and to detect local epidemiological priorities.

These data were continually collected and then analyzed monthly and annually by Project staff for monitoring of coverage of health indicators and of the epidemiological situation. The community health data gathered were shared regularly (usually quarterly) with the community at assemblies (*asambleas*) to discuss progress, celebrate achievements, address gaps and challenges, and engage in problems as well as to fully engage in improving their own health. Each community had a *sala situacional* (“situation room”) – a public space where the community’s health data were exhibited in easily understood graphic form – to stimulate interest, awareness, and transparency.

#### Application of the Care Group Approach in the Project Area

A Care Group field guide was used as a reference [52]. Mothers selected by the community served as peer educators called Care Group Volunteers to encourage healthy behaviors and the appropriate utilization of health services. Care Group Volunteers were often non- or semi-literate.

**Fig. 5:**
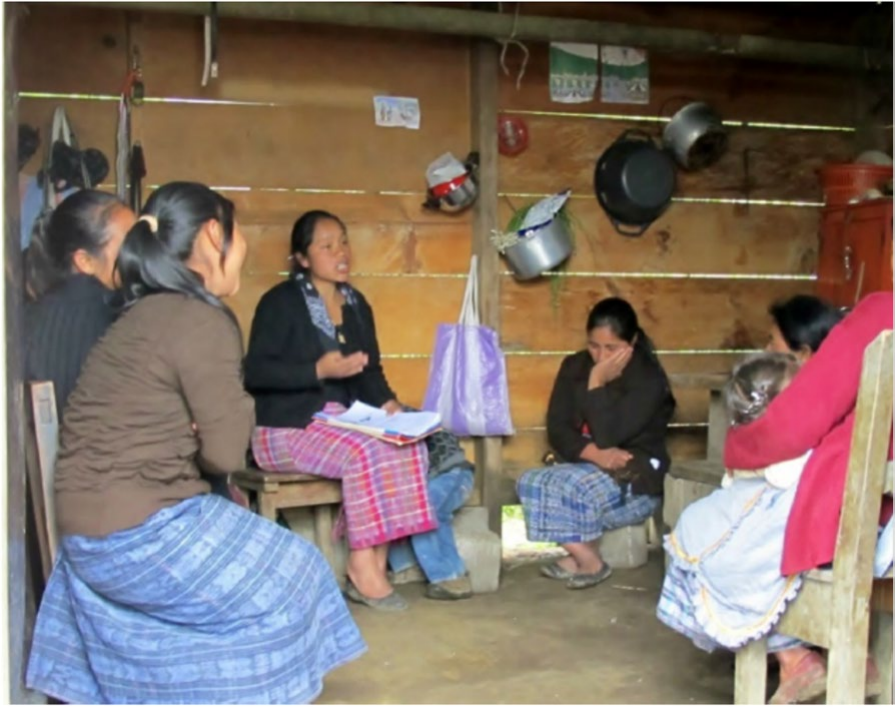
A Care Group meeting: A Level-1 Care Group Promoter (*Facilitadora Comunitaria*) leading a training session with Care Group Volunteers (*Comunicadoras en Salud*) in her home. Source: Ira Stollak

**Fig. 6:**
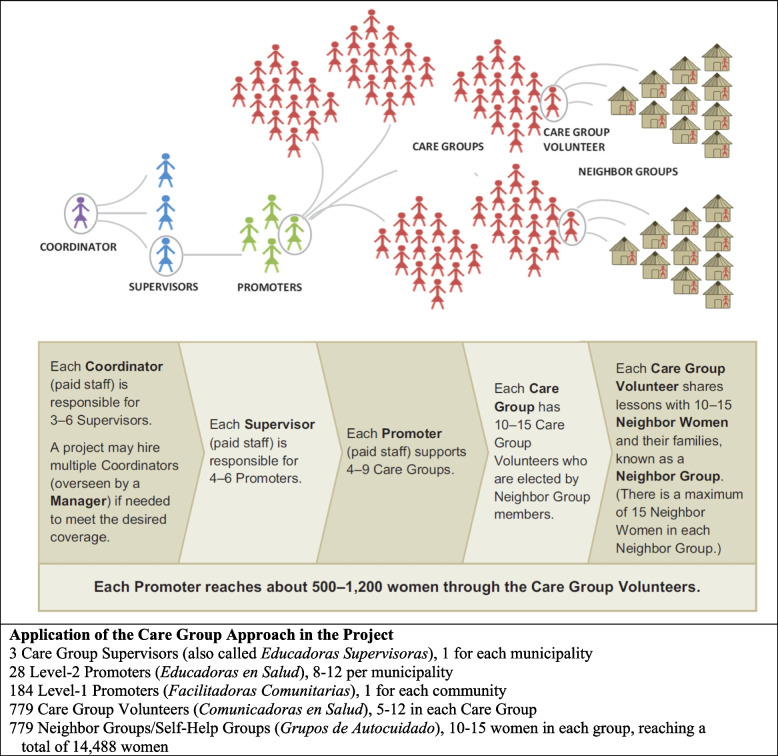
The Care Group training cascade and its application in the Curamericas/Guatemala Child Survival Project. Source: Figure obtained from Perry et al. [53]

Each of the Project’s 28 Health Educators (*Educadoras en Salud*, referred to here and in subsequent papers in this series as Level-2 Promoters) trained one Community Facilitator (*Facilitadora Comunitaria*, referred to subsequently as Level-1 Promoters*)* in each community (184 in all), who in turn trained one or two Care Groups with each consisting of 5–12 mother peer educators/Care Group Volunteers known as Health Communicators (*Comunicadoras en Salud*) (Figs. 5 and 6). There were 779 Care Group Volunteers in all.

Each Care Group Volunteer carried out twice-monthly meetings with 10–15 mothers of under-2 children who had been assigned to them. These neighbor women were organized into Self-Help Groups (*Grupos de Autocuidados*). The Care Group Volunteer met with their Self-Help Group and also, between meetings, visited the homes of the individual members of the Self-Help Group. In each of the three municipalities there was a Care Group Supervisor (*Educadora Supervisora*) who trained and supported the Level-2 Promoters in his/her municipality.

To avoid confusion and to encourage comparison with other applications of the Care Group Approach in other settings, we will use the standard terminology for these persons as contained in the published description of the Care Group Approach [53] and as shown in Fig. 6.

The Level-1 and Level-2 Promoters and the Care Group Volunteers used participatory learning techniques for non-literate adult audiences to teach key life-saving messages such as, but not limited to, the need for antenatal and postnatal care; the recognition of and prompt response to danger signs during pregnancy, delivery, and the post-partum period; the recognition of and correct response to symptoms of childhood pneumonia and diarrhea; the importance of exclusive breastfeeding during the first six months of life and proper complementary feeding thereafter; the importance of child immunizations; and point-of-use water treatment along with proper hand washing at critical moments.

Care Group Volunteers were also responsible for detecting and reporting vital events among their assigned Self-Help Group women, including new pregnancies, births, and deaths, thus establishing a community-based vital events surveillance system as part of the CBIO Approach. The Care Group Volunteers detected these vital events either in the Self-Help Group meetings or during home visits and conveyed the information to their supervising Level-1 Promoter at the time of the subsequent Care Group meeting. The Level-1 Promoter, in turn, passed this information to her﻿ supervising Level-2 Promoter during their twice-monthly trainings and she in turn reported this information to her Care Group Supervisor (*Educadora Supervisora)*, who consolidated the vital events data from all her Level-2 Promoters and passed them on to the Institutional Facilitator for the municipality. As shown in Fig. 3, an Institutional Facilitator Supervisor supported the Municipal Institutional Facilitators and aggregated their municipal vital events data into Project-wide vital events analyses and reports.

In addition, the timely detection and reporting of pregnancies and births by the Care Group Volunteers led to the prompt provision of services to pregnant and puerperal women and newborns. Throughout their pregnancy, newly pregnant women were monitored for complications by the Level-1 and Level-2 Promoters and referred for prenatal care at the Birthing Center (see below) or the PEC program (see below). Post-partum women were referred for post-partum checks to the same sources of care. All reported deaths were followed up within two weeks with a verbal autopsy conducted by the Institutional Facilitator (who was a nurse). The completed verbal autopsies were reviewed for quality assurance by the Institutional Facilitator Supervisor to ensure quality vital events data.

Curamericas/Guatemala developed a manual of Care Group lessons covering all the Project’s targeted health behaviors and indicators [64]. The manual contains a year-long cycle of lessons that were taught in the local language and included ice-breakers, learning games, songs, skits, practicing of skills (such as breastfeeding and hand washing) and testimonials. The lessons embraced the learners’ capacity for theoretical understanding and taught concepts such as germ theory, nutritional content of food, the principles behind immunizations, as well as the reasons for antenatal care, health facility deliveries, and post-partum care. Another key aspect of the pedagogy was that it involved “just-in-time” learning: the learners in the training cascade taught others below them in the cascade soon after they had been taught in order to minimize recall error. For example, the Level-1 Promoter (*Facilitadora Comunitaria*) taught her Care Group Volunteers in the same way the Level-2 Promoter (*Educadora en Salud*) taught the Level-1 Promoter (and within a week while the lessons were fresh), using the same learning materials. This “just-in-time replication” flowed rapidly down the entire training cascade to help ensure fidelity and quality.

#### Application of the Community Birthing Center Approach in the Project Area

The strategy of Birthing Centers staffed with Maya-speaking health professionals providing culturally adapted and appropriate services in the local language was developed by Curamericas/Guatemala in the Project Area prior to 2011. The application of the Birthing Center Approach is based on guidelines that are contained in a field manual [65]. The Birthing Center is not a maternity waiting home (where women come from far away, await the onset of labor, and deliver their baby adjacent to a referral facility rather a locally available facility where women come at the onset of labor to deliver their baby with the assistance of a skilled, trained health worker and where a rapid response transport capability is available should a complication arise.

During the Project’s implementation period, there were three Birthing Centers in operation: one in Calhuitz (municipality of San Sebastian Coatán), which opened in 2009; one in Santo Domingo (municipality of San Sebastián Coatán), which began operation mid-way through the Project in 2013, and one in Tuzlaj-Coya (in the municipality of San Miguel Acatán), which began operation during the final year of the Project, in 2014.

Each of the three Birthing Centers was staffed by an auxiliary nurse and two support women (*mujeres de apoyo*), who were trained and supported by an obstetric nurse supervisor who was based in Calhuitz. This maternal care program included training and integration of traditional birth attendants (*comadronas)* as well as the establishment of an emergency response system to transport women to the Birthing Center and, if necessary, from the Birthing Center to the nearest referral hospital. The auxiliary nurses were trained by the obstetric nurse supervisor in safe deliveries and in Essential Newborn Care, which included clean cord care, immediate thermal care, and immediate/exclusive breastfeeding; the Active Management of the Third Stage of Labor, including the use of partographs (a system for monitoring the progress of labor and for identifying possible complications) and the administration of oxytocin after the delivery of the baby; as well as neonatal resuscitation (using Ambu bag and mask) and stabilization/resolution of any neonatal complications.

The referral system included radio telephones and transportation linkages to emergency medical technicians (who were located at the neighboring town of San Antonio Huista outside of the Project Area at a lower elevation) to transport women and neonates to the nearest hospital in the city of Huehuetenango, about four hours away (depending on the location of the Birthing Center). Birthing Center services were free of charge and provided in the local Maya language; local birth customs were respected.

The Project staff encouraged their clients to deliver in the Birthing Center, as previously reported [60]. A growing number of women utilized a Birthing Center; their *comadronas* accompanied them there and assisted appropriately in the delivery. Qualitative research conducted by three of us (BL, KL, NM) through individual and structured group interviews with *comadronas* revealed a unanimous positive attitude toward the Birthing Centers for two main reasons: (1) the staff at the Birthing Centers made them feel welcome and a valued part of the care team, and (2) the *comadronas* realized that if a complication arose, the mother would receive better medical care than if the delivery were taking place in the home [66].

Gradually, the Birthing Centers have become places where additional services have been provided beyond the delivery of babies. They provide health education, antenatal care, postnatal care, and treat children with symptoms of pneumonia and severe diarrhea. They also organize and hold meetings for support groups (*círculos)* for pregnant women (*círculos de embarazadas*), for lactating women (*círculos de madres lactantes*), and adolescent girls (*círculos de adolescentes*)*.* Although these operate independently of the Care Groups and Self-Help Groups, women may participate in multiple activities. In 2014 the Birthing Centers were equipped with small, self-sustaining pharmacies (*boutiquines*) through a partnership with Medicines for Humanity. This enabled the Birthing Centers to provide antibiotic treatment for infections and other basic primary health care treatments (e.g., for rashes, scabies, or minor infections).

An additional activity that the Birthing Centers took on was training and support for *comadronas* in collaboration with the MSPAS. The MSPAS has long been struggling to define the role of the *comadrona* in the rural health system and settled on a “harm-reduction” approach that involved the training of *comadronas* by MSPAS staff in the provision of clean and safe births and home-based life-saving skills to improve the safety and quality of their home deliveries. Curamericas/Guatemala has been a long-term collaborator with the MSPAS in the provision of this training, dating back to 2002. Later, the *comadronas* were﻿ integrated into the Birthing Center team as described above.

The Birthing Center*s* were designed for cultural acceptability. The physical structure of the Birthing Center is based upon the design of traditional Maya homes. In addition to an exam room, delivery room, and post-partum recovery room, a Birthing Center includes a traditional Maya kitchen where the woman’s family can prepare traditional food, and a *chuj*, the traditional Maya sweat lodge. Services are provided in the local Maya language and local birth-related customs are respected. To strengthen cultural acceptability and the utilization of the Birthing Center, the local *comadronas* were integrated into the Birthing Center team: they brought women in labor to the Birthing Center to deliver instead of attending the deliveries by themselves in the women’s homes. The *comadronas* assisted the Birthing Center staff appropriately in the delivery.

The Birthing Centers were designed to ensure accessibility. They each serve a catchment of 8–12 communities known as a micro-region, which is a set of communities that choose to engage with and support the establishment of a new Birthing Center*.* These communities are located within 8–10 km of their Birthing Center*.* Services were always available 24 h a day, 7 days per week, with Birthing Center staff on-call in rotating shifts.

The Birthing Centers were based on the CBIO principles of community engagement and community partnership. The communities within a micro-region established a Micro-Regional Committee composed of representatives from each community. These representatives were trained to manage the construction of the Birthing Center and its operations once it began functioning. The Birthing Center was built and maintained entirely with volunteer community labor on land donated by the municipal government with materials provided by both the communities and the Project. The catchment communities making up the micro-region are referred to as “partner communities” and communities that are not located in a micro-region are known as “non-partner communities.” Women from any non-partner community were free to use any Birthing Center. Also, to enhance women’s participation, a Woman’s Support Committee was established for each Birthing Center*,* each undertaking an activity to enhance their Birthing Center’s services (e.g., a kitchen garden of traditional medicinal herbs at the Santo Domingo Birthing Center).

The Birthing Centers are financially accessible. Services are free, whether the woman was from a partner or non-partner community. There was a small optional fee of approximately US$7, which was for food and cleaning of linens. Alternatively, the families could provide these themselves. Pregnant women and their families also had the option of making a one-time payment of approximately US$7 to the Micro-Regional Committee as insurance to assist with the cost of emergency transport to the hospital if needed. If the insurance fee was paid, one-half of the US$150 cost of transport to the referral facility (the MSPAS hospital in the city of Huehuetenango) was paid by the Micro-Regional Committee. Most of this fee covered the cost of the ambulance service of the emergency medical technicians in the town of San Antonio Huista, approximately an hour drive away, who, after being contacted by satellite phone by the Birthing Center staff, received the patient at a point approximately half-way between San Antonio Huista and the Birthing Center and then transported her the rest of the way (approximately three hours) to the MSPAS hospital in Huehuetenango. The balance of the fee was used to pay the local on-call service provider (usually someone nearby who had a mini-van) who brought the patient from the Birthing Center to the rendezvous point with the emergency medical technicians. While the approximately US$75 net cost to the insured family of the transported woman was high in this context of poverty, families of women in need of emergency transport managed to gather this sum.

#### Implementation of the government’s Extension of Coverage Program (PEC)

The national *Programa de Extensión de Cobertura* (PEC) was established in 1996 based on the early experience with a pilot program in the Project Area in which the Project Director (Dr. Mario Valdez) played a leadership role. PEC was established to strengthen primary health care and extend health services to rural Indigenous communities. In essence, PEC sent mobile nurses into communities on a monthly basis to provide primary health care. The government contracted with local NGOs throughout Guatemala to operate PEC in specific geographic areas. In two municipalities (San Sebastián Coatán and San Miguel Acatán), Curamericas/Guatemala had a contract with MSPAS to operate the PEC program on its behalf. In one of the Project municipalities (Santa Eulalia), an NGO by the name of ADIVES (*Asociación de Desarollo Integral de Vida y Esperanza*) implemented PEC under contract with MSPAS.

The MSPAS clinics were distant from most of the communities and difficult to access due to poor roads, mountainous terrain, and the cost of transport in both money and time. PEC made it possible for ambulatory/mobile nurses to provide primary health care in each community, with a focus on maternal and child health. Each nurse served 10–15 communities and visited each community on a fixed date once per month to provide primary health care services, including antenatal and post-partum checks, iron/folate supplementation, and tetanus vaccinations for pregnant women; vitamin A supplementation and deworming for children, childhood immunization, child growth monitoring, family planning, as well as treatment and follow-up for sick children.

The PEC ambulatory nurses came to a strategic location in each community. In some communities this was a small building owned by the MSPAS with basic medicines and equipment, called a Health Post, which was unlocked for services when the PEC nurse arrived. In other communities they met in another type of building such as school. These locations were called *centros de convergencia.* Due to national political upheaval, the government abruptly terminated the PEC program just as the Project was beginning its final year of operations, in October 2014. As we discuss in other papers in this supplement, this led to an abrupt cessation of PEC services, including immunization, vitamin A supplementation, antenatal care and family planning services in the Project Area along with treatment of childhood illnesses, which Curamericas Project staff were not authorized to do.

#### Summary of services provided by the integrated rural primary health care system

High-impact interventions delivered by the Project included: quality antenatal care; health facility deliveries; timely post-partum care; Essential Newborn Care; Active Management of Third Stage of Labor; proper hand washing, water purification and point-of-use water treatment; safe water storage and feces disposal (by means of a latrine away from the house and from water sources); proper treatment and care-seeking for childhood diarrhea and pneumonia; immediate post-partum breastfeeding; exclusive breastfeeding during the newborn’s first six months of life along with proper complementary feeding during the 6–<24-month period; and promotion of childhood immunizations and family planning. These interventions thus combined (1) achieving sustainable behavior change at the household level (e.g., hand washing and exclusive breastfeeding) primarily via the Care Groups, with (2) the promotion and provision of geographically and culturally accessible health services at community-based health posts (conducted by mobile nurses provided by the PEC program) and at Birthing Centers (where not only deliveries took place but also where antenatal care was provided, breastfeeding support groups met, and children with pneumonia were treated)*.*

## Discussion

CBIO+ is a methodology for strengthening community-based primary health care that builds on time-honored principles of community partnerships, routine systematic visitation of all homes, addressing both locally defined epidemiological priorities as well as health priorities that are defined by the community, establishing women’s support groups, and incorporating local cultural traditions when possible, including traditional midwives. Many, but not all, of these principles have been embedded in the implementation of many other effective and sustainable long-term programs as small-scale projects that became the basis for scaled-up large scale programs.

CBIO principles have been implemented in pioneering programs throughout the world, most notably the icddrb Maternal and Child Health/Family Planning Project in Matlab, Bangladesh [67–69], the Hospital Albert Schweitzer in rural Haiti [70–72], the Jamkhed Comprehensive Rural Health Project in central India [73, 74], and SEARCH (Society for Education, Action and Research in Community Health) in central India [75, 76]. These projects are also notable because of their long-term effectiveness in reducing mortality in children [77].

Basic elements of CBIO+ have been implemented at scale, including census-based approaches to routine systematic home visitation in Bangladesh [78–80], Brazil [81], Nepal [82] and Ethiopia [83], among others. Ethiopia, for instance, has implemented a national program of regular visitation of homes by community health workers that has made the country a leader in Africa for reducing under-5 mortality; controlling HIV, tuberculosis and malaria; and expanding access to contraception [84, 85]. Care Groups have been established in numerous countries throughout the world [53], and local Birthing Centers have been established in the urban slums of Bangladesh [86]. However, the Curamericas/Guatemala Maternal and Child Health Project is the first example of which we are aware that assesses the effectiveness of CBIO+ as a combined, comprehensive approach to strengthening community-based primary health care and improving health and well-being in a geographically defined population at the level of a district.

The Project faced notable challenges in the implementation of CBIO+ , not the least of which were difficulties in accessing homes because of the steep terrain and lack of roads along with the presence of multiple dialects within the Project Area.

## Conclusion

The Curamericas/Guatemala Maternal and Child Health Project, 2011–2015, implemented by Curamericas/Guatemala with support from Curamericas Global, built on previous experiences with programming to improve maternal and child health in the Western Highlands of the Department of Huehuetenango, Guatemala. The Project integrated three promising approaches to foster community ownership, community engagement, and reduce mortality through high population coverage of key maternal and child health interventions: (1) the Census-Based, Impact-Oriented (CBIO) Approach, (2) the Care Group Approach, and (3) the Community Birthing Center Approach*.* The Project built partnerships with the Guatemalan Ministry of Health, its Extension of Coverage Program (PEC), the municipalities (districts), community leaders, and the community members themselves – especially the women. The Project aimed to create an equitable and comprehensive local health system that integrated communities with the local health system. Subsequent articles in this supplement describe the implementation research and the findings related to the effectiveness of the Project in improving the health and well-being of mothers, children, and communities.

## Data Availability

All of the Project reports, de-identified data, as well as publications about the Expanded CBIO+ Approach cited in this article are available from the corresponding author on request.

## Abbreviations

ARHC: Andean Rural Health Care
CBIO: Census-Based, Impact-Oriented
CBIO+: The Expanded CBIO Approach (joint implementation of the CBIO Approach together with Care Groups and Community Birthing Centers called *Casas Maternas Rurales* as a single Project strategy)
KPC: Knowledge, practice, and coverage (household survey)
M&E: Monitoring and evaluation
MMR: Maternal mortality ratio
MSPAS: *Ministerio de Salud Pública y Asistencia Social* (Ministry of Public Health and Social Welfare)
PEC: *Programa de Extensión **do** Cobertura* (Extension of Coverage Program)
